# Nature in the Office: A Systematic Review of Nature Elements and Their Effects on Worker Stress Response

**DOI:** 10.3390/healthcare11212838

**Published:** 2023-10-27

**Authors:** María Luisa Ríos-Rodríguez, Marina Testa Moreno, Pilar Moreno-Jiménez

**Affiliations:** Department of Social Psychology, Social Work, Social Services and Social Anthropology, University of Málaga, 29016 Málaga, Spain; marinatesta@uma.es (M.T.M.); mpilar@uma.es (P.M.-J.)

**Keywords:** biophilic design, stress, worker, systematic review

## Abstract

Work-related stress is a significant problem in many work environments and can have negative consequences for both employees and organisations. This review aimed to identify which elements of biophilic design in the workplace affect workers’ stress response. To enable this, a literature search was conducted using PsycINFO, Scopus, and Medline. The search was limited to articles published from 2012 to June 2023. This review only integrated quantitative data, incorporating twelve records for qualitative synthesis. The selected studies suggest that strategies such as access to outdoor environments or the creation of outdoor areas are effective in reducing stress in the workplace. If these are not feasible, the examined research advocates the use of virtual means to recreate such relaxation or break spaces. Furthermore, aspects of interest for future research were identified, such as multisensory stimulation, including the sense of smell, the exploration of views with natural elements, the creation of shelters, or the study of biomorphic forms.

## 1. Introduction

Awareness of the importance of mental health in the workplace has led to the identification of emerging psychosocial risks, among which work-related stress has been recognized as one of the most pressing challenges in terms of occupational health and safety. [1]. As for its definition, work-related stress encompasses the physical and emotional response resulting from the mismatch between work demands and the resources and capabilities perceived by individuals to cope with those demands [2].

Simultaneously, reports such as the one presented by the WHO [3] have highlighted the relevance of workplaces as spaces of influence on mental health, urging the implementation of strategies that ensure psychological well-being, such as psychosocial risk prevention and promoting well-being in the workplace. This correlation between adverse working conditions and mental health repercussions is supported by empirical evidence and underscores the need for interventions that directly address this issue, especially in the wake of the recent health crisis [2,4,5]. In order to address this challenge, studying the interactions between contact with nature and its effects on mental health is an encouraging and growing perspective [6,7]. According to Markevich et al. [8] the potential pathways linking natural spaces to health emphasize three functions: damage reduction, capacity restoration, and capacity development. Consequently, understanding which forms of contact with nature have a greater impact on mitigating work-related stress can provide specific guidelines for the planning and design of work environments, offering benefits that will be addressed below.

### Benefits of Biophilic Design

In the field of environmental psychology and occupational health, increasing attention has been paid to the influence of urbanization and the reduction in contact with nature [9,10]. Frameworks like the attention restoration theory, ART [11] and stress recovery theory, SRT [12] have been useful for analysing the effects of nature on health and assessing the implications of living in built environments. ART focuses on how natural and restorative environments can help restore attentional capacity and alleviate accumulated mental fatigue. According to ART, sustained attention in urban and highly stimulating environments can lead to attention fatigue and reduced cognitive performance. In contrast, exposure to natural environments, such as parks or green areas, provides an opportunity for engagement in fascination-driven attention, allowing for cognitive rest and the recovery of attentional fatigue [13,14,15,16,17]. This theory highlighted how the presence of natural elements, such as vegetation and water, can provide an environment that facilitates the disconnection from cognitive demands and the restoration of attention. In this vein, numerous studies have focused on exploring restorative experiences in built environments [18,19,20,21]. Stress recovery theory (SRT), is focused on the immediate reaction to environmental stimuli. SRT suggests that exposure to nature and natural elements can trigger positive physiological and emotional responses, including the reduction of sympathetic nervous system activity associated with stress [22]. Furthermore, the theory indicates that seeing and interacting with nature can promote recovery from mental and emotional fatigue, which in turn contributes to greater satisfaction and relaxation [23].

In this context, it is worth noting that an approach that aims to reconnect occupants with the natural environment is known as biophilic design. This movement is based on the idea that humans have an innate and profound connection with nature, and the integration of natural elements into built spaces can have a significant impact on psychological well-being, physical health, and the quality of life for individuals [24,25]. Hence, there have been numerous attempts to identify patterns that serve as a guide to direct designers’ practices. One of the most recent proposals is that of Browing et al. [26] where up to fourteen patterns were identified (Table 1). In this field, the effect of aspects such as thermal comfort, natural lighting, or views on workplace productivity have been verified [26,27,28,29].

In this context, we detected a significant increase in research on the effects of nature contact on health and well-being [7,30,31,32]. Among the benefits of this connection, Frumkin et al. [33] pointed out an improvement in sleep quality, a reduction in anxiety, depression, stress, and aggressive behaviours, as well as an increase in the perception of happiness, well-being, and life satisfaction. In this regard, biodiversity has been one of the influential factors in stress recovery [34]. Likewise, in an integrative review of thirty studies, significant associations were demonstrated between nature exposures and improvements in mood and the reduction of stress [35]. Among their contributions, the authors pointed out that it is necessary to document and specifically identify those elements of nature used in the research, as well as the experiences carried out in natural areas. In this way, it facilitates urban design professionals in applying this knowledge to urban reality and improving public health.

In turn, another line of research examined the effect of exposure to virtual nature. In a recent review of 59 studies [13], it was observed that virtual nature was beneficial in reducing perceived stress, as well as in physiological indicators such as heart rate (greater relaxation) and results in electroencephalograms (associated with relaxation levels and cognitive recovery). Other indicators such as electrodermal activity, blood pressure, cortisol, or salivary amylase showed inconsistent findings, thus requiring further research.

Despite the accumulation of evidence regarding the beneficial impact of nature on the management of psychophysiological stress, whether through exposure to nature (real or virtual) or through engagement in activities in green spaces [36,37,38,39], this knowledge has barely been transferred to workplace contexts.

In this regard, we find studies about indoor environmental quality, confirming that such indoor environmental quality is predictive of workers’ productivity in office settings [40]. Similarly, literature review studies, such as the one by Colenberg et al. [41] have confirmed the relationship between office interior design and physical, psychological, and social well-being. Their findings underscore the significance of individual control and the inclusion of real or artificial plant elements in the design of healthy workspaces, although they do not delve extensively into these aspects. Likewise, another study focused on the use of natural elements indoors, particularly the use of wood, which emphasizes this material’s ability to positively influence stress indicators and highlights the need for further research in this field [42]. This combination of research studies highlights the need to further explore how the inclusion of natural elements and the application of biophilic principles can enhance the well-being and mental health of workers in contemporary workplace environments.

In conclusion, work-related stress is a significant issue in many workplace environments and can have negative consequences for both employees and organizations. The evidence suggests that contact with nature can have a positive impact on stress reduction, but it is important to systematically examine and evaluate the available scientific literature to draw more robust conclusions.

When conducting a systematic review, the aim is to systematically gather and rigorously analyse relevant studies that have investigated the effects of nature contact on the reduction of work-related stress. By specifically addressing various types of biophilic design patterns, the goal is to understand which specific elements have a greater impact on stress reduction. This can provide practical and concrete information for employers and workplace designers.

## 2. Methods

### 2.1. Literature Search

A literature review was conducted to describe current knowledge about biophilic design interventions on office employee stress. The PRISMA statement was used as a reference for its development. Thus, comprehensive searches were carried out in the *PsycINFO*, *Medline*, and *Scopus* databases between 2 July and 9 July 2023. Database titles were identified and used in combination with specific search terms. Each search term was used first separately and then combined with Boolean operators. The search terms and combinations are presented in Table 2. In this context, specific terms were incorporated to pinpoint positions within the realm of biophilic design. Subsequently, during a screening phase, the ones that resonated with this objective were chosen. The search limits were set to English and Spanish languages. No additional restrictions related to study design or intervention duration were imposed. The timeframe for publications was established from 2012 to June 2023. Furthermore, manual searches were conducted to supplement the database of studies in the following journals: *Journal of Environmental Psychology, Journal of Architectural and Planning Research, Journal of Environmental Psychology and Sustainable Development, Journal of Applied Psychology, Journal of Organizational Behavior*, *Journal of Occupational Health Psychology and Work & Stress*.

### 2.2. Inclusion and Exclusion Criteria

Regarding the inclusion criteria, it was established that the studies should be empirical, excluding grey literature. The second criterion was that the sample should consist of office workers; therefore, studies focused on students were excluded. Third, research on biophilic design in the workplace was included. These interventions should have as an essential feature contact with nature, either directly or virtually. Since we were interested in psychological and psychophysiological outcomes, articles published in journals classified in the fields of sciences (mathematics, physics, chemistry, materials science, etc.) were excluded.

Regarding the outcome variables, studies that assessed stress, burnout, or anxiety were included, both through self-report measures and physiological indicators. Since the interest was to understand the effect on occupational stress, articles focused on clinical aspects, such as depression, post-traumatic stress, generalized anxiety disorders, etc., were excluded. All other bibliographic outcomes that did not evaluate stress or were focused on different effects fell outside the scope of this review. Examples of excluded outcomes were the following: stress risk factors (workload, conflicts, job support), coping skills, changes in knowledge or attitudes, job performance, and/or staff turnover.

### 2.3. Data Extraction

**Figure 1:**
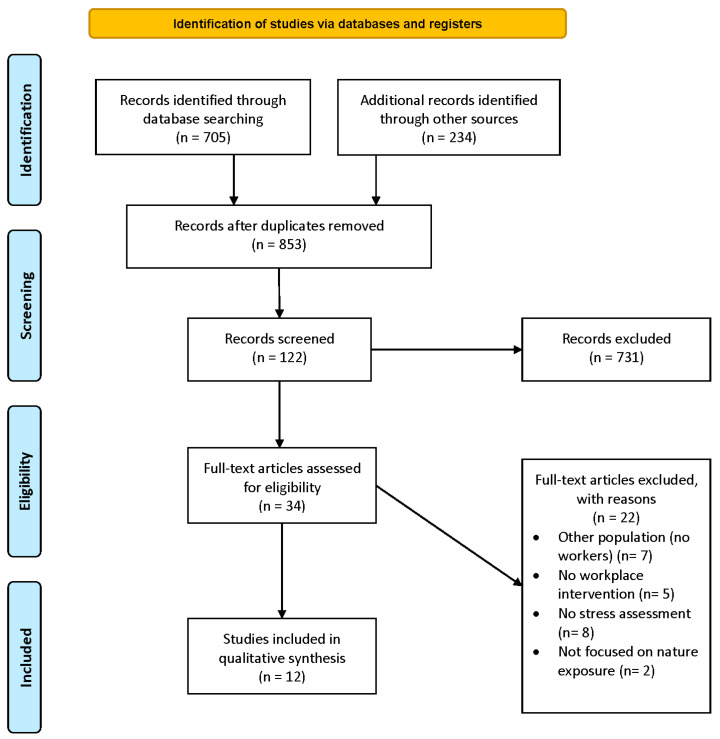
PRISMA 2020 flow diagram for new systematic reviews, which included searches of databases and registers only.

After the initial searches, 705 potentially eligible studies were identified from the database results, and 234 were found through manual searches. The removal of duplicates resulted in 853 relevant reports. Titles and abstracts were reviewed, and full-text articles were obtained from potentially eligible titles. In cases of doubt, discussions were held among the authors. Figure 1 shows a flowchart illustrating the article inclusion process.

Titles and keywords were examined based on the inclusion criteria, leading to the exclusion of 731 studies. In a second phase, abstracts were reviewed, yielding 34 articles that met all inclusion criteria. These works were examined, and 20 were excluded for reasons such as: (1) not using a sample of office workers; (2) not conducting the intervention in the workplace; (3) not specifically evaluating stress; (4) focusing on the physical space in general rather than the nature component. Finally, eligible records were identified, and their full texts were downloaded. From this last step, 12 records were included for qualitative synthesis.

## 3. Results

### 3.1. Characteristics of Included Studies

The characteristics of the included studies are summarized in Table 3. First, it was observed that six of the studies were conducted in the United States, four in Europe (Finland, Sweden, the United Kingdom, and Norway), and two in Asia (Japan and South Korea).

Regarding the study design, five were observational studies. Within this category, three used a cross-sectional survey design [43,44,45], one was an analytical observational study with a prospective cohort design [46] and the other was a longitudinal repeated measures study [47]. The remaining seven studies were experimental or quasi-experimental in nature, with three conducted in laboratory conditions [48,49,50] and four field studies conducted in workplace settings [51,52,53,54]. Regarding the participants, all studies sampled office workers, except for two that included other samples [44,52]. In most of the observational studies, the sample size exceeded one hundred participants, and none exceeded 570. However, in the experimental studies, the sample sizes did not exceed forty participants, except for the study by Douglas et al. [48].

Regarding the use of biophilic design, two studies exclusively employed technological means (video projections, playback of natural sounds, virtual reality, etc.) [49,50], while another two combined real natural elements (plants, candles, water features, etc.) with the use of technology [46,53]. Four research works focused solely on exposure to natural elements [48,51,53,54]. Finally, three of the studies used self-report measures to assess the perception of biophilic design [43,45,47].

Likewise, it was observed that in all the studies, some measure of stress was included as an output variable, and additionally, some studies included anxiety, burnout, and/or arousal. In this regard, a single instrument for measuring stress was not found; we only found it in two studies [46,54], that used *The State-Trait Anxiety Inventory* (STAI), while two others used single-item measures [48,49], the remaining eight studies used other instruments (Table 2). In addition to these measures, five studies collected some physiological indicators for stress assessment [46,48,49,50,54].

Similarly, other output variable constructs were found beyond the variables of interest considered in this review, with the most common ones being positive and negative affect [46,47,49] and creativity [48,50]. In the same vein, we also found a study that included the assessment of vitality [49]. Furthermore, within environmental psychology, some of the variables we observed included a connection with nature, environmental satisfaction, environmental concern, restorativeness, and belongingness. Likewise, more closely related to occupational and organizational psychology, we also assessed organizational support, job satisfaction, organizational citizenship behaviour, somatic complaints at work, and work absenteeism due to illness.

### 3.2. Main Effects

Having detailed the peculiarities of the examined studies, we now proceed to independently analyse the results of these investigations. Beginning this exploration, it is imperative to note that six of the selected studies provided concrete evidence regarding the benefits derived from the implementation of breaks in the workplace and the consequent reduction in stress associated with these rest periods. In this context, it is relevant to highlight that the typical time frame considered in these studies ranged from 10 to 15 min, except for the studies conducted by Yin et al. and Largo Wight et al. [50,54], where a 3 min interval was used.

Regarding how to optimize these breaks with the inclusion of nature, one of the examined topics was whether there were differences between resting in indoor or outdoor spaces. In this regard, the results were inconsistent. Bjørnstad et al. [43] noted in a cross-sectional survey study that indoor nature contact was significantly associated with lower job stress, subjective health complaints, and sick leave. However, no significant relationship was found between outdoor nature contact and job stress. In contrast, Largo-Wight et al. [51] found through a randomized controlled trial that taking a 10 min break outdoors significantly reduced stress compared to taking these breaks indoors. This result is consistent with the study by Perrins et al. [47] in which a longitudinal repeated measures study showed that spending more time in outdoor environments was associated with lower anxiety (state) scores, regardless of the activity or location. However, indoors, both location and type of activity influenced anxiety levels.

For example, indoor environments such as the workplace and home also contribute to reducing anxiety levels. It is concluded that natural outdoor environments, by themselves, have a beneficial effect. Another observational study in this line was conducted by Lottrup et al. [45] where they found a reduction in perceived stress levels in two self-reported conditions related to the patterns *Connection with Natural Systems* and *Visual Connection with Nature*—when there is physical access to green outdoor environments or when the workplace has views of green outdoor environments, with a caveat that this result was only significant in the male sample but not in the female sample. Finally, Yin et al. [50] through virtual reality exposure, confirmed that, in general, the effects of biophilic interventions on reducing physiological stress were consistently better in open spaces.

On the other hand, an attempt was made to determine which type of sensory stimulation is most beneficial for stress reduction. To answer this question, we focus on the following two studies. Aristazabal et al. [46] created simulated open office environments, establishing a control group and three experimental conditions, which were as follows: (1) visual, where they introduced indoor plants, digital projections, and artwork with nature scenes throughout the office space; (2) auditory, where they included reproductions of stimuli such as wind, dripping water, streams, or sounds produced by regional wildlife where the study was located, in addition to a real sound-producing water fountain; (3) multisensory, where a multisensory combination of visual and auditory biophilic components was presented. Among the conclusions they obtained, a reduction in sympathetic nervous system (SNS) activity was found in the visual and multisensory conditions. Specifically, one of the measures (NS-SCR amplitude, related to skin conductance) suggested lower stress in the multisensory condition. This result was also observed in participants’ self-reported stress assessments, where the greatest stress reduction occurred in the multisensory condition. Similarly, the study by Ojala et al. [49] differentiated three experimental conditions, (1) and (2), where they combined virtual video environments and sounds (one with forest environments and the other with water environments), and (3), where participants were exposed to an auditory environment—a neutral room without any stimulation served as the control group. Among the conclusions they reached, they noted that all breaks (including the control break, consisting of a silent interval) resulted in reductions in stress levels, but multisensory conditions were the most effective in this regard. Finally, it is worth noting that only in the study by Putrino et al. [52] were olfactory elements included in the spaces created for contact with nature.

Regarding the use of biophilic design patterns, the fundamental question was whether patterns of nature in the space, patterns of natural analogies, and nature space patterns produced differences in the stress levels of workers. In this regard, we highlight the results provided by Roskam and Haynes [53] who emphasized the indirect evocation of nature as one of their study objectives. To conduct this, they designed regeneration capsules that imitated the refuge dimension (within the nature space patterns), the use of wood and biomorphic design (within the nature analogues pattern), and the option to recreate natural soundscapes. Stress levels, anxiety, and mental load were compared between different time points during work (where cognitive performance tasks were proposed). Among the conclusions of the study, it is worth noting that the regeneration capsules used were effective in reducing anxiety to a greater extent than meeting rooms, both immediately (after use) and in a second round of performance tasks. This result provides greater flexibility in conducting biophilic design interventions in workplaces. In the same vein, the work of Douglas et al. [48] indicated that finishes in natural materials (such as wood or stone) can be a more cost-effective biophilic design measure and can favour the reduction of stress, both negative excitement and physiological activation. Likewise, self-reported stress measures were lower in rooms with natural materials. Additionally, in the category of natural analogy patterns, the authors reported a smaller increase in stress when experimental rooms introduced diverse iconography (racially diverse individuals, mainly women) compared to the inclusion of non-diverse iconography (represented only by white men).

The rest of the studies focused on incorporating patterns of nature in the space. Specifically, studies on the use of plants and windows (associated with views of nature and access to natural light) as elements that enhance indoor office spaces and have an effect on reducing stress. However, the use of windows in the study by Douglas et al. [48] resulted in no self-reported stress measures. Regarding plants, a specific study on the effects of including small potted plants on the work desk revealed reductions in state anxiety scores for men (with this reduction not replicating in women). In the same vein, the use of green decoration elements, green features, green spaces, and natural light were associated with better stress resilience [44].

**Table 3:** Characteristics of included studies.

Authors/Year	Country	Sample	Aim & Design	Type of Nature	Outcome Variable(s)	Results
Aristizabal et al., 2021 [46]	USA	Office workers (n = 35)	Prospective cohort design to examine the effect of nature on occupant experience in a simulated, open-office environment (Time: 8 weeks) *Condition 1 (control*): Baseline office environment with no environmental aspects. *Condition 2.* Experimental condition with biophilic visuals. *Condition 3.* Experimental condition with biophilic sounds introduced to the office space. *Condition 4.* Conditions 2 and 3 described above in addition to a water feature.	A multisensory biophilic environment, as opposed to an environment with solely visual or auditory elements.	*Physiological indicators* of stress (such as Heart rate, Skin Conductance Level) *Subjective measures* - Stress (The Job Stress Scale, Lambert et al., 2007 [55] - Anxiety (STAI), Spielberger et al., 1983 [56] - Affects - Connectedness to nature - Satisfaction with environmental features *Environmental measurements* *Cognitive performance measures*	Exposure to biophilic elements in all three conditions decreases SNS reactions. All biophilic conditions showed a positive effect on participants’ workplace stress although marginally less job stress in the visual condition. Self-ratings of stress were consistent with these results.
Bjørnstad et al., 2016 [43]	Norway	Employees in seven public and private offices (n = 565)	Cross-sectional survey to investigate whether contact with nature at work is associated with employee health and engagement and the mediating role of perceived organizational support.	Nature Contact Questionnaire (NCQ)*,* Largo-Wright et al., 2011 [57] outdoors during working hours, indoor nature contact	*Psychological measures* - Job stress (Job Stress Survey, JSS-N) Spielberg et al., 1999, 2004 [58,59] - Perceived organizational support - Subjective health complaints and sick leave.	More indoor nature contact at work was significantly associated with less job stress, fewer subjective health complaints and less sickness absence. Perceived organizational support mediated the associations between contact with nature indoors and work stress and sickness absence, and partly mediated the association with subjective health complaints. Outdoor nature contact did not show a reliable association with the results of this study.
Douglas et al., 2022 [48]	USA	Staff, faculty, graduate students (With some amount of professional experience) *Pilot study* (n = 272) *Experimental* (n = 413)	Test the biopsychosocial effect of certain physical characteristics in simulated work environments. (1) Pilot study: online survey (2) Experimental lab study	Materials (natural vs. artificial) Windows or no windows Representations and iconography (diverse or no diverse)	*Physiological indicators* - Analysis of Skin - Conductance Responses *Psychological measures* - Belonging - Creativity - Environmental concern - Stress: Single item Karvounides et al. 2016 [60], valence and arousal Mauss and Robinson, 2009 [61]	In the pilot study, a reduction in self-reported stress was observed with the presence of natural materials and diverse representations. The windows also significantly reduced self-reported negative arousal. In the experimental study, it was observed that in this condition exposure to natural materials significantly decreases self-reported stress. No differences in self-reported stress were observed with the window stimulus.
Han et al., 2020 [44]	South Corea	Hotel employees (n = 280)	Cross-sectional survey to study the use of eco-design to reduce stress among service employees in the hotel sector	Items on green décor, green items, green space and natural light	- Stress resilience (ad hoc) - Emotional exhaustion - Job satisfaction - Organizational citizenship behaviour	They validated a structural equation model showing that a green design increases resilience to stress (0.451 **) and its relationship to the other variables in the study.
Largo-Wight et al., 2017 [51]	USA	University office staff (n = 244; n = 36)	The aim was to study the feasibility and reliability of the outdoor booster break (OBB) to analyse the effect of OBB on stress levels. *Phase 1:* Online survey *Phase 2:* A single-site randomized controlled trial (RCT) (Period: 4 weeks). *Control group*: OBB indoor *Treatment group*: OBB outdoor	Outdoor booster break (OBB) indoor vs. outdoor.	- Stress: The Perceived Stress Questionnaire (PSQ) Levenstein et al., 1993 [62]	The outdoor work break protocol was perceived as worthwhile, practical, and feasible. The outdoor booster break reduced stress significantly more than an indoor break. A main effects ANCOVA model controlling for baseline stress revealed that post-test stress was lower for the treatment group compared to controls.
Lottrup et al., 2013 [45]	Sweden	Workers (n = 439)	A cross-sectional survey to investigate whether access to a green outdoor environment at work is related to employees’ perceived level of stress and attitude toward the workplace. *WG-Index 1* No view (green outdoor environment); no physical access to any outdoor environment *WG-Index 2*. View of a green outdoor environment; no physical access to an outdoor environment dominated by greenery *WG-Index 3*. Physical access to an outdoor environment dominated by greenery	Workplace greenery Index (3 conditions)	- *Level of stress*. Perceived stress, irritation, and fatigue. (EQ-VAS, which is a subscale of EuroQoL, Brooks & De Charro, 1996 [63], Brooks, Rabin, & De Charro, 2003 [64] - *Workplace attitude*.	The results show that there are differences according to the gender of the participants. In women, stress levels are higher and the relationship between workplace vegetation was not significant. In contrast, in men, there is a relationship between stress and workplace vegetation. Finally, the results indicate that physical access to workplace vegetation has greater benefits than purely visual access.
Ojala et al., 2022 [49]	Finland	Full-time employed participants (n = 39)	Experimental design to study the effects of taking breaks in a virtual natural environment on stress recovery (Period: 9 sessions with different conditions). A: Forest (video + audio) B. Water (video + audio) C: Sound (audio) *Control:* Exposure to silence.	*Experimental:* exposure to virtual natural environments in three conditions.	*Psychological measures* - Restorative - Positive and negative affects - Vitality - Anxiety. Marteau and Bekker’s, 1992 [65] - Baseline work stress *Physiological measures* Heart rate variability (HRV)	All pauses, including control (silence), have stress-relieving effects, but a multi-sensory experience reduces stress better than presenting only audio or visual material. This recovery is observed by a greater restoration and a decrease in heart rate. Breaks with virtual natural environments contribute to decreased anxiety and increased parasympathetic nervous activity.
Perrins et al., 2021 [47]	USA	Amazon workers Study 1 (n = 153) Study 2 (n = 33)	The aim of the study was to analyse how characteristics of workers’ day-to-day environments may impact mental health outcomes like affect, depression, and stress. Two studies are presented: (1) A cross-sectional survey; (2) Longitudinal assessments (with stratified sampling) (Period: 2 weeks).	*Study 1*. Hypothesis: more frequent visitation to the Spheres (multistorey nature conservatories) would be associated with lower anxiety and stress (and others). *Study 2*. Longitudinal assessments of psychological well-being and degree of naturalness.	*Study 1:* Nature contact and Trait relatedness to nature - Affects and activity type - Depression, anxiety and stress Lovibond and Lovibond, 1995 [66]. *Study 2:* Positive affective - State anxiety (State-Trait Anxiety Inventory-Short Form, Marteau and Bekker, 1992 [65] - Participants’ current location - Activity type	*Study 1* More self-reported frequency of visitation to the Spheres was significantly associated with more positive affect and less negative affect in the base models, but these associations were no longer statistically significant when controlling for various activities. *Study 2* Time spent in more natural environments is associated with less state anxiety in outdoor settings, even after taking activity and location into account. Within indoor environments, the significant relationship between environment naturalness and state anxiety was reduced and no longer significant with location and activity.
Putrino et al., 2020 [52]	USA	Frontline healthcare workers (n = 219)	Quasi-experimental study to verify if the use of recharging rooms with a biophilic design decreases the perceived stress in first-line healthcare workers (Period: 14 days)	Recharge Room with multisensory design (visual, auditory, and olfactory), and nature-inspired experiences.	Item measurements were taken regarding the level of perceived stress before and after the experience. Measure of user experience and optional “additional comments”.	After a single 15 min experience in the Recharge Room, the average user-reported stress level was significantly reduced.
Roskam & Haynes, 2020 [53]	UK	Employees private company (n = 32)	A randomized field experiment to analyse the effectiveness of biophilic “Restoration pods” in promoting recovery from stress. *Control:* condition involving a 10 min break in an enclosed meeting room. *Treatment:* A condition involving a 10 min break in the regeneration pod.	Regeneration pods using bamboo wood and designed to follow the structural logic of nature using complex biomorphic forms and sounds of nature.	*Subjective measures*: - Stress (The anxiety-comfort subscale of the multi-affect indicator, Warr, 2013 [67] - Perceived mental demand, temporal demand, perceived effort. - Tasks performance - Performance in proofreading and arithmetic task	The regeneration pods were more effective at reducing stress and anxiety than the meeting room. Office spaces do not have to incorporate biophilic ‘nature in space’ design strategies (e.g., plants or direct views of nature) to be restorative. Indirect evocation of nature (materials, biomorphic forms, and sounds of nature) is also beneficial.
Toyoda et al., 2020 [54]	Japan	Office workers, a private company (n = 63)	A field experiment to test whether a small plant on the desk has the potential to reduce stress. Phase 1. Control (one week, without plants) Phase 2. Intervention (two weeks, to learn how to care for plants, two weeks, to care for plants independently).	Small plants on the desk	*Psychological measurement* Psychological stress STAI-Form JYZ, Hidano et al., 2000 [68] A self-completed open-ended questionnaire *Physiological indicators* Pulse rates	A significant decrease in STAI scores after the intervention phase. However, this difference was not significant in women. Of the participants 58.7% did not show significant changes in pulse rate. STAI scores and changes in pulse rate were not significantly related. There were neither differences by age, nor by type of plant in the scores in STAI, or in the physiological measures.
Yin et al., 2019 [50]	USA	Students and staff from the Harvard T.H. Chan School of Public Health (n = 30)	A randomized crossover study with three versions of biophilic design in simulated open and enclosed office spaces in virtual reality (VR).	Simulating three types of biophilic design interventions (i.e., natural elements, natural analogues and combo)	*Physiological indicators* - Blood pressure - Heart rate - Heart rate variability - Skin conductance level *Cognitive outcomes* - Reaction time and creativity. *Covariates* - Caffeinated beverage drinking - Sleep quality before the experiment day - The self-reported stress level before the experiment.	Participants in three spaces with biophilic elements had consistently lower levels of physiological stress indicators. These effects varied according to the type of workspace (open or closed), with open biophilic spaces having a greater reduction in physiological stress. In terms of differences according to the three types of biophilic exposure, the participants who showed the lowest levels of stress were those in the “natural elements” condition.

** *p* < 0.01.

## 4. Discussion

In the context of this review, our objective was to identify and synthesize the body of scientific publications on the effects of biophilic design on stress in workplace environments. Due to the heterogeneity in the design of primary studies, conducting a meta-analysis did not appear to be a recommended option. Instead, we focused our efforts on the concise extraction of information from the various research papers.

In this context, it is important to consider the mode of contact with biophilic design that was used. In two cases, the interaction was exclusively virtual, in three, self-report measures were used that were not linked to exposure, while in the others, real contact was combined with virtual exposure. In relation to this, White et al. [69] emphasized that there is a widespread preference for contact with nature in the real world, although the use of virtual reality can be a valid alternative. Specifically, exposure to nature has a positive impact on stress reduction and promotes relaxation [70,71,72].

Regarding the design of experimental or field study situations, we observed that scheduled breaks in these types of work generally had a duration of between 10 and 15 min, although in some cases, they were shorter. This finding aligns with previous research on the optimal duration of breaks. Albulescu et al. [73], in their review and meta-analysis, concluded that work breaks with a minimum duration of 10 min are more effective. These breaks not only alleviate fatigue and increase energy levels but also tend to improve work productivity.

Regarding the differences between resting in indoor or outdoor environments, with the exception of a single study, all the examined research supports the benefits of outdoor spaces. These findings align with previous findings in the workplace context [57,74,75,76]. However, the ability to simulate outdoor environments through virtual reality emerges as a valuable alternative when access to natural spaces is limited. In this context, a solid body of evidence has accumulated supporting the positive effects of virtual nature in reducing negative emotions, psychological restoration, and reducing the perception of stress, as well as in certain physiological stress-related indicators [36,77]. Additionally, researchers have highlighted potential benefits in terms of positive emotions, vitality, creativity, and perceived safety, aspects that still require further investigation and detailed examination [78,79]. These findings are aligned with Ulrich’s restoration theory [22], which also highlights the physiological and emotional benefits associated with nature exposure.

Another aspect we identified was the effect of using different modalities of sensory stimulation. Most studies focused on visual and auditory stimuli or a combination of both. However, only one study [52] incorporated olfactory elements into their research. It is important to note that, in general, the impact of odours in urban environments has been relatively understudied, despite the informative potential they can offer [80]. In an experimental study that used virtual reality exposures by [81], they tested the hypothesis that natural environments such as forests or parks, involving multisensory stimulation (visual, auditory, and olfactory), may facilitate greater stress reduction compared to urban areas. The results highlighted that odour had a more significant effect on stress response compared to auditory and virtual stimuli. Additionally, it was found that the degree of psychological liking experienced with these odours in parks and forests was associated with lower levels of physiological stress, a result that was not observed with the other two senses. Therefore, including olfactory stimulation in workplace environments is a promising research avenue. This result, along with the preference and the positive effect of contact with nature, supports the attention restoration theory (ART) [11], where reference is made to the effect of this contact on attention and, consequently, on cognitive restoration.

Regarding biophilic design patterns, in the nature in the space pattern, the analysed studies indicate that the inclusion of plant elements tends to produce consistent effects on stress reduction, especially when self-report measures are used [43,44,48]. These findings align with previous research that also suggests a physiological reduction in stress response in the general population. For example, a meta-analysis focusing on the effects of indoor plants found that they can have notable benefits, such as reducing diastolic blood pressure [82]. Furthermore, previous reviews focused on forest therapy support this trend by showing decreases in heart rate and blood pressure not only through direct exposure but also through the use of audiovisual resources such as videos [83]. Therefore, the inclusion of green elements in workplace environments or direct contact through breaks in green spaces can be considered an effective strategy for reducing stress.

As for the *nature analogues* pattern, one key finding relates to the impact of using natural materials such as stone and wood in reducing stress in workplace environments. This observed benefit in work contexts aligns with a systematic review conducted by Lipovac and Burnard et al. [42] who emphasized the potential of wood in building construction, although they advised that caution is required before concluding that it affects physiological stress indicators, both due to methodological issues and the rigour of the studies. In any case, this line of research is of special interest as it provides additional elements that are not necessarily green in workplace environments, thus offering more flexibility in designing these spaces.

In addition to studies focusing on the use of wood, Roskam and Haynes [53] explored, along with this dimension, the use of biomorphic forms when creating restoration capsules. These capsules included an aspect related to the nature in the space pattern, involving the creation of refuge spaces. These capsules provided workers with a place to concentrate, allowing them to observe their surroundings from a privileged space. These experiences of indirect evocation of nature are of particular interest due to their ability to offer a more relaxing and productive work environment. Therefore, we consider it an emerging research area in the workplace context. At the same time, we noticed that there are very few studies addressing the relationship between views of natural elements from windows and stress in workplaces, even though both variables appear to be related and are associated with high work performance and job satisfaction [84,85].

### 4.1. Strengths and Limitations

In this review, an exhaustive literature search was conducted in three databases, revealing that various terms are used in the field of biophilic design, which complicates the task of refining the search for articles specifically focusing on workplace environments and their effects on stress. In this regard, we consider one of the strengths to be the application of exclusion criteria, such as the evaluation of a single variable (stress) rather than broader aspects (such as well-being), which address multiple components. This choice is based, among other reasons, on the ability to measure stress through self-reports or physiological indicators, which can enrich and strengthen the conclusions.

However, the evaluation itself lacks coherence regarding the instruments to be used and the physiological measures to be considered in research. This inconsistency reduces the possibility of replicating studies and generalizing conclusions. The use of disparate self-reported measures does not allow for comparisons between studies and verifying if the evidence is replicated. Regarding physiological measures, they may provide inconclusive results since there are few, and as Lipovac and Burnard [42], have stated, different measures of autonomic arousal can function independently or even in opposition to each other in response to specific affective states, such as feelings of interest. Therefore, it is important to be cautious in this regard. Additionally, only two of the reviewed studies incorporated a stress-inducing activity to interpret more accurately changes in arousal levels. Lastly, despite several of the reviewed studies indicating the need for longitudinal research to examine contact with nature in workplace environments, only one of the selected studies [47] conducted the said type of research and it was a study of short duration (2 weeks).

Regarding the characteristics of the studies, there is also notable diversity in terms of the locations where these investigations were conducted. These differences encompass not only different countries but also continents, resulting in variations in cultural dimensions and values in each territory. Therefore, the studies cannot be considered homogeneous in this regard.

In addition to geographic and cultural diversity, differences in sample sizes were observed, with unknown statistical power in some cases. In this methodological context, it is relevant to highlight the disparity in approaches in study design. First, field studies entail costs that affect the control of variables, raising the question of complementing them with laboratory research that isolates the analysed aspects and provides a stable environment. An essential aspect in this regard is the inclusion of samples of office workers. It is important to mention that several studies excluded from this review focused on university students, a situation that we consider should not be assumed to be comparable. Although in some cases, this may be valid for knowledge workers who spend a considerable amount of time in office environments, the differences in objectives between educational and workplace organizations make this issue one that should be carefully considered.

### 4.2. Practical Recommendations

After analysing the selected studies, it can be inferred that exposure to contact with nature is more effective when it is real, although virtual alternatives also offer benefits. It is suggested that future research or interventions should focus on evaluating the impacts of direct contact with nature in workplace environments. This involves incorporating natural elements such as plants and materials like wood or stone. Additionally, exploring improvements in space design for revitalization is proposed. In this regard, given the potential implementation limitations in workspaces, it is recommended to direct efforts towards creating accessible outdoor areas or specific spaces for revitalization. For the latter option, it is advisable to create multisensory conditions that engage sight, sound, and smell, along with the application of biomorphic forms in the design.

### 4.3. Future Lines of Investigation

Studies on the contributions of biophilic design in the workplace seem to have been more focused on predicting well-being than on preventing adverse effects such as stress. In this regard, one of the issues raised by one of the studies examined in this review was the null effect of windows [48]. Generally, this design element has been associated with improvements in well-being, whether in natural spaces or constructed environments [86] and with one of the less explored patterns (nature of the space) that may be related to the need for fascination. Consequently, we believe that one necessary future research direction is to investigate the effect of having window views on stress, taking into account their characteristics.

Similarly, future research needs to consider individual aspects, such as the differences based on gender. In addition to workplace-related factors, such as the impact of smart work on the arrangement and design of home environments, the integration of advanced technologies and flexible work setups in our living spaces has the potential to significantly influence how we perceive and use our homes for professional activities. Regarding the study designs, there is also a need to conduct longitudinal studies to verify the long-term changes resulting from contact with natural elements.

## 5. Conclusions

The conclusions drawn from this study are of importance in assessing the impacts of biophilic design in the workplace, a context that has not been examined sufficiently despite being the place where we spend the most time. First and foremost, its relevance is highlighted because workplace contexts, especially those related to office jobs, generally take place indoors. From this perspective, promoting access to outdoor environments or creating outdoor relaxation areas emerged as two strategies that have been effectively applied. Alternatively, the research examined advocates for the use of virtual means to create these spaces for disconnection or breaks.

On the other hand, this study identifies future research directions that are yet to be addressed in the workplace context regarding stress, such as multisensory stimulation, including the sense of smell, investigating views of natural elements, creating sheltered spaces, or studying biomorphic forms. Additionally, it underscores the potential influence of the gender variable as a covariate, emphasizing the pressing need to deepen our understanding of the differences that may arise in terms of how biophilic patterns impact the stress levels experienced by office workers.

In summary, this study constitutes a significant contribution to the existing body of knowledge by examining the intersection between biophilic design and stress in the workplace. It provides practical and applicable recommendations for employers and workplace designers on how to effectively incorporate natural elements to reduce workplace stress.

## Figures and Tables

**Table 1:** Patterns of biophilic design.

Type	Patterns
Nature in the space	Visual Connection with Nature
	Non-Visual Connection with Nature Data
	Thermal and Airflow Variability
	Presence of Water
	Dynamic and Diffuse Light Connection with Natural Systems
Natural analogues	Biomorphic Forms and Patterns
	Material Connection with Nature
	Complexity and Order Prospect
Natures of the space	Refuge
	Mystery
	Risk/Peril

**Table 2:** Search terms/combination.

#1	“workplace” OR “workplaces” OR “office” OR “worksite health promotion” OR “work” OR “office buildings” OR “workspace” OR “healthcare workers” OR “employees” OR “staff”
#2	“biophilia” OR “biophilic design” OR “physical environment” OR “natural environment” OR “nature connectedness” OR “connectedness to nature” OR “nature contact” OR “indoor plants” OR “plant” OR “plants” OR “views” OR “windows view” OR “nature exposure” OR “green” OR “green space” OR “blue space” OR “garden” OR “visual connection with nature” OR “Non-visual connection with Nature” OR “presence of water” OR “sensory information”
#3	“stress” OR “stress recovery” OR “work stress” OR “symptoms of stress” OR “stress employee” OR “employee mental health” OR “work-related stress” OR “stress reduction” OR “burnout” OR “psychological stress” OR “job stress” OR “occupational stress”
#4	#1 AND #2 AND #3 Title or abstract

## Data Availability

Not applicable.
